# Epitope tags are not created equal: Disruption of cellular function of a translation factor by a short viral tag

**DOI:** 10.17912/micropub.biology.001452

**Published:** 2025-02-18

**Authors:** Brooke Barker, Chloe Cannon, Hannah Umphlett, Eun Suk Kim, Brett D Keiper

**Affiliations:** 1 Biochemistry & Molecular Biology, Brody School of Medicine, East Carolina University, Greenville, North Carolina, United States

## Abstract

Cellular identity and fate are determined by the proteins synthesized. Initiation of mRNA translation requires an important translation factor, eIF4G ( *ifg-1* in *C. elegans* ). Embryos use mRNA translational control for spatial and temporal regulation of protein synthesis. Using CRISPR engineering, we added in-frame epitope and fluorescent tags (V5, Myc, Flag, GFP, and mCherry) to IFG-1 . Tagged forms containing the V5 epitope caused embryonic arrest. Internal disruption of the V5 tag restored viability at 25°C. This study demonstrates that the molecular nature of a small epitope tag is sufficient to disrupt *C. elegans* embryogenesis.

**Figure 1. The effect of tags on the function of eIF4G:**
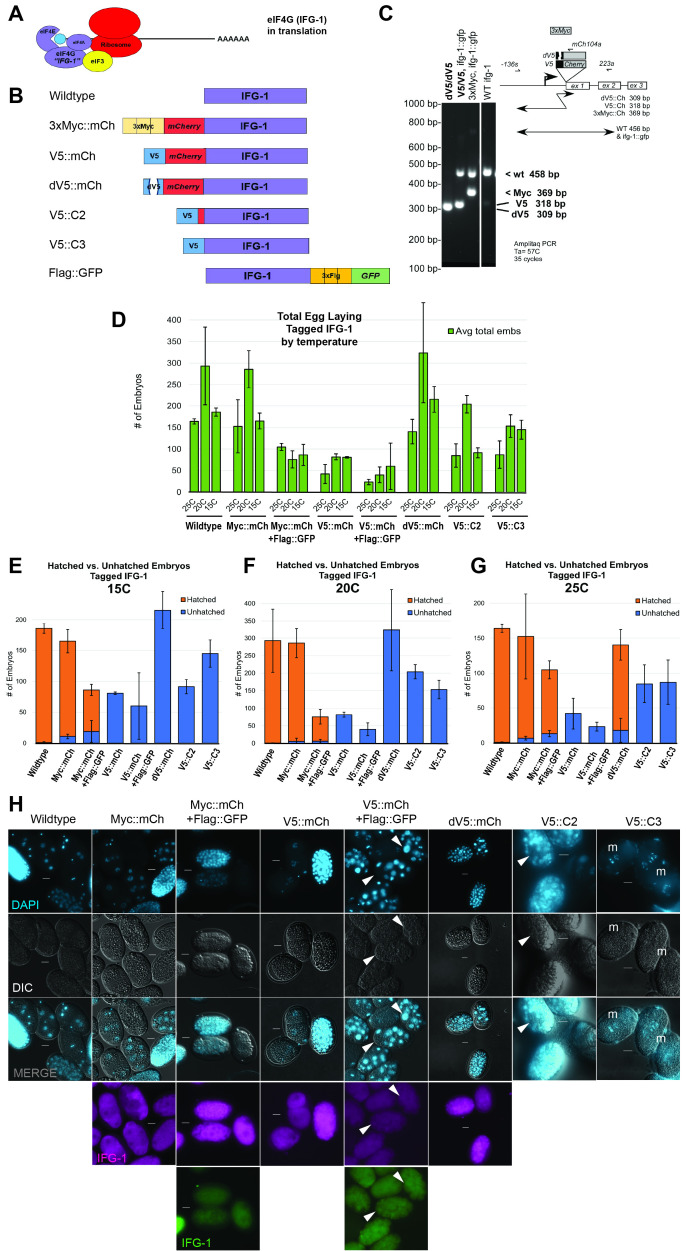
(A) A diagram of the translation initiation complex assembled on eIF4G. (B) Various gene constructs engineered for IFG-1 (eIF4G) used throughout this study. Wildtype has no tags. Alternate tagged forms contain in-frame fusion of 3x Myc fused to mCherry (3xMyc::Ch), a V5 epitope tag fused to mCherry (V5::Ch), a V5 tag with an internal three amino acid deletion fused to mCherry (dV5::Ch), a V5 tag and small spacer without mCherry (V5::C2), V5 fused directly adjacent to IFG-1 (V5::C3), and an integrated transgene encoding 3x Flag fused to GFP on the IFG-1 C-terminus (Flag::GFP). (C) Single worm genomic PCR with various primer sets (shown) to distinguish endogenous (CRISPR) and exogenous *ifg-1* gene fusions in selected strains. (D) Total counts of eggs laid (green bars) by a single hermaphrodite for each of the various genotypes (x-axis) at each incubation temperature 25°C, 20°C, and 15°C. Error bars represent the standard deviation. Two strains co-express both mCherry and GFP-tagged versions of IFG-1 (Myc::Ch + Flag::GFP and V5::mCh +Flag::GFP). (E-G) Assay of egg hatching as a measure of embryo viability. Stacked bars indicate the number of hatched viable embryos (orange bars) to unhatched embryos (blue bars). Graphs are derived from the same data set shown in (D) but depict their ability to hatch and represent separate temperatures. The results represent three biological replicates. (H) Fluorescence imaging of embryos raised at 20°C from each IFG-1 engineered strain. DAPI staining (first row); DIC (second row); DAPI plus DIC channels merged (third row); mCherry in magenta (fourth row); GFP in green (fifth row). Each column (#1-8) displays images corresponding to the construct(s) labeled above. For embryos that could not hatch (V5::mCh, V5::mCh + Flag::GFP, dV5::mCh, V5::C2, and V5::C3) the terminal phenotype is displayed. White arrows point to regions of cells lacking stainable DNA or with abnormally large nuclei. “m” represents metaphase-arrested chromosomes evident in V5::C3 only. Scale bars = 10 μm. Images were taken at 40x (wildtype, Myc::mCh, Myc::mCh + Flag::GFP, V5::mCh, V5::mCh + Flag::GFP, and dV5::mCh) or 100x (V5::C2 and V5::C3) magnification.

## Description

The *in vivo* tagging of proteins using CRISPR engineering is important for assessing localization, function, and for purification purposes (Kim *et al.* 2014). We genetically modified the eIF4G gene in *C. elegans* ( *ifg-1* ) to natively express red (mCherry) or green fluorescent (GFP) with epitope-tags (V5, 3x Myc, and 3x Flag) ( Fig. 1B, C ). The translation factor eIF4G plays a vital role in scaffolding the initiation complex which recruits ribosomes to mRNA (Hentze 1997; Gray and Wickens 1998; Keiper *et al.* 1999; Prevot *et al.* 2003) ( Fig. 1A ). If any interaction during mRNA translation becomes disrupted, protein synthesis may suffer. Since protein synthesis is a central metabolic function required for embryonic development, aberrant synthesis may interfere with embryogenesis (Keiper 2019) . We questioned whether additions of tags *per se* affect eIF4G's native function or cellular localization.

Fertility was classified by evaluating the number of embryos laid by mothers of each genotype at 25°C, 20°C, and 15°C. Among the tagged forms, the 3xMyc tag fused to mCherry demonstrated minor disruption to fertility and no substantial loss compared to normal development ( Fig. 1D ). The same genotype, but with additional co-expression of IFG-1 ::Flag::GFP, showed a significant decline in fertility ( Fig. 1D ). Similarly, all of the V5-tagged forms (with or without fluorescence) laid an abnormally low number of embryos. However, a 3 amino acid deletion (Leu-Gly-Leu) within the V5 tag (dV5) restored fertility of mothers to a great extent—most significantly at 20°C (dV5::mCh, Fig. 1D ). When addressing viability of embryos, we again observed that the Myc::mCherry tag has the least detrimental impact on eIF4G function for hatching; this is evident by nearly normal fertility and viability across all temperatures ( Fig. 1E-G ). Although the addition of Flag::GFP tagged IFG-1 to the Myc::mCherry version displayed lower fertility rates, these embryos were largely viable to hatch. All tags containing the V5 epitope presented a 0% hatching rate at all temperatures, arresting prior to hatching ( Fig. 1E-G ). Co-expression of IFG-1 ::Flag::GFP exacerbated the V5 loss of fertility. Remarkably, a small deletion in V5 unexpectedly restored offspring number (Fig 1D). Embryo viability was surprisingly also restored, but only at 25°C ( Fig. 1G ). Therefore, in addition to rescuing fertility—even above wildtype levels at 15°C and 20°C, disruption of this tag can restore all embryonic functions of IFG-1 . The loss of Leu-Gly-Leu from the largely hydrophobic V5 tag (Pro-Ile-Pro-Asn-Pro-Leu-Leu-Gly-Leu-Asp) may marginally decrease its hydrophobic nature but does not prevent recognition by an antibody directed at the V5 tag.

Embryos of the Myc genotypes (Myc::mCh and Myc::mCh + Flag::GFP) show normal division of embryos from the 4-cell out to the ~100-cell blastula ( Fig. 1H, columns 2, 3). In all cases of V5-tagged versions, many nuclei appeared to be variable in size and distribution ( Fig. 1H, arrowheads). Depending on configuration, the V5 tag showed arrests at various stages up to the ~100-cell stage ( Fig. 1H ). To specifically observe the toxic effect of the V5 tag itself, we analyzed constructs including V5 but without fluorophore (V5::C2, V5::C3). These slightly differing constructs showed embryonic phenotypes of aberrant nuclear location and size ( Fig. 1H, arrowheads). The most extreme case was shown for V5::C3 where chromosomes display a metaphase arrest and halt development already at the 2-cell stage ( Fig. 1H, “m” symbol). Among all V5-tagged IFG-1 proteins (4 genotypes), the directly adjacent V5 epitope resulted in the most lethality to development ( Fig. 1H, columns 7, 8). Under non-rescue conditions of 20°C, the internally deleted V5 tag gave similar results of embryonic arrest (~100 cell stage) as V5-tagged forms ( Fig. 1H, column 6). Despite the embryonic arrest, however, we see no major disruption of expression or localization of mCherry or GFP fluorescent IFG-1 protein ( Fig. 1H, rows 4 and 5).

Most tags fused to IFG-1 had little deleterious effects on fertility or embryo viability. It was therefore surprising that the commonly used V5 epitope tag resulted in universal disruption of embryo development when fused to IFG-1 in any context. Curiously, that maternal effect embryonic lethal phenotype does not match an *ifg-1 ( ok1211 )* null phenotype, where worms arrest as L2 larvae (Contreras *et al.* 2008). Our data shows that longer tags (e.g. 3xMyc::mCherry adds about 25% length and mass) do not have broad deleterious effects on development. In contrast, any construct containing the V5 tag diminished not only fertility but also the ability of eggs to hatch, i.e. embryonically lethal ( Fig. 1E-G ). The exception to this was the dV5 genotype, specifically at 25°C, which largely rescued fertility, embryonic viability and larval/adult worm development ( Fig. 1G ). Indeed, these worms remained viable for multiple generations at 25°C. However, the toxic effects of intact V5 on embryos were quite nuanced. In particular, embryo cleavage varied greatly among the V5-tagged forms, with the most severe case being V5 fused directly to IFG-1 (V5::C3) which showed a 2-cell metaphase arrest. Other configurations of V5 exhibited aberrant nuclear distribution and size ( Fig. 1H, arrows). This suggests an advantage of having “buffer” sequence between IFG-1 and the V5 tag; embryos arrested later in development ( Fig. 1H ). Therefore, adjacent fusion of V5 to IFG-1 is most detrimental to development. A recent report noted that a small (Flag) or large (GFP) N-terminal tag on lamin equally alters its subnuclear localization and its activity (Odell and Lammerding 2024) . Our findings indicate that all tags are not created equal. Interference with IFG-1 function does not correlate with the tag size or proportion, but rather with the nature of the amino acid sequence added. Small tags (e.g. viral derived V5 tag) have the potential to cause severe detrimental effects while large protein fusions at the same position are fully tolerated. We propose that V5 itself is disruptive to the function of eIF4G. It may block normal mRNA translation and compromise embryogenesis. This study suggests that caution should be used in assessing a protein's function based on small tag fusions.

## Methods

Genetic engineering of *C. elegans* using CRISPR-Cas9 allowed us to introduce tags into the *ifg-1* gene (Klionsky *et al.* ). Small antigenic tags [viral V5 (15 amino acids), internally disrupted V5 (12 amino acids), 3x Myc (31 amino acids)] in addition to the mCherry coding region (252 amino acids) on the N-terminal end of IFG-1 (1156 amino acids). Gene constructs and repair templates were planned in DNAStar Lasergene (v17.5). Repair templates and guide RNAs (gRNA) ordered from Integrated DNA Technologies (IDT) as Alt-R™ HDR donor oligos or created by PCR with similar 5' end modifications. Guide RNAs were designed using the IDT CRISPR design site ( https://www.idtdna.com/site/order/designtool/index/CRISPR_SEQUENCE , RUO22-1364_001). A transgene encoding 3xFlag::GFP (278 amino acids) added in frame to the C-terminal end of the *ifg-1* gene was injected into wild type worms as a complex array and UV integrated and outcrossed 8 times to HT1593 (obtained from C. elegans Genetics Center, University of Minnesota); the integration site was not determined. Selection was done by introduction of red or green fluorescence and/or PCR of F1 progeny pools ( Fig. 1C ). All CRISPR and integrated strains were outcrossed 1-5 times and combined by further genetic crosses (see strain list below). Embryonic lethality assays were performed using homozygous L4 hermaphrodites (“mothers”) to lay eggs. Mothers were separately placed on “seeded” agar plates and allowed to grow at various temperatures, i.e. 15°C, 20°C, and 25°C; transferal to secondary plates facilitated progressive counts. Mothers were collected after egg laying, frozen, lysed, and genotyped by PCR, conducted as previously described (Contreras *et al.* 2008). Laying was observed in 24-hour periods on a stereo-dissecting microscope. Hatching was assayed after laying was complete for >24 hours at the appropriate temperature during a second observation. Embryo and hatchling counts were evaluated from 48-240 hours and assayed in triplicates. Embryo and hatchling counts were compiled by time and date, then graphed in Excel. To perform imaging, adults were dissected to obtain fresh embryos, which were fixed in 3% formaldehyde, then 70% ethanol and stained with DAPI. Embryos were mounted on 2% agar pads and imaged using a Zeiss Observer 7 fluorescent microscope. Images were processed in ZEN 2.3 blue edition software (Carl Zeiss Microscopy, GmbH).

## Reagents

**Table: Worm strains used in the study**

**Table:** 

Strain Name	Genotype	Source
Wild type	N2	Caenorhabditis Genetics Center
HT1593	*unc-119 ( ed3 ) III *	Caenorhabditis Genetics Center
KX182	*ifg-1 ( eu22 [V5::mCherry:: ifg-1 ])/ mIn1 [ mIs14 dpy-10 ( e128 )] II *	Keiper lab
KX187	*ifg-1 ( eu22 [V5::mCherry:: ifg-1 ])/ mIn1 [ mIs14 dpy-10 ( e128 )] II* , *euIs(pPD ifg-1 ::gfp, pAZ81, Cb unc-119 +) *	Keiper lab
KX188	*ifg-1 ( eu23 [V5::17aa:: ifg-1 ])/ mIn1 [ mIs14 dpy-10 ( e128 )] II* ; {V5::C2}	Keiper lab
KX189	*ifg-1 (eu24[V5::6aa:: ifg-1 ])/ mIn1 [ mIs14 dpy-10 ( e128 )] II* ; {V5::C3}	Keiper lab
KX212	*ifg-1 ( eu32 [3xMyc::mCherry:: ifg-1 ]) II, unc-119 ( ed3 ) III, euIs[pPD ifg-1 ::gfp, pAZ81 Cb unc-119 (+)] *	Keiper lab
KX214	*ifg-1 ( eu22 [deltaV5::mCherry:: ifg-1 ])/ mIn1 [ mIs14 dpy-10 ( e128 )] II *	Keiper lab
KX228	*ifg-1 ( eu32 [3xMyc::mCherry:: ifg-1 ]) II *	Keiper lab
